# An airbag cap specially designed for the removal of sharp, deeply embedded foreign bodies: a new method of cap-assisted endoscopy

**DOI:** 10.1055/a-2109-0991

**Published:** 2023-07-13

**Authors:** Linlin Chen, Zhaoxia Lu, Yuanyuan Zhou, Miao Ouyang

**Affiliations:** 1 The Fourth Department of the Digestive Disease Center, Suining Central Hospital, Suining, Sichuan, China; 2 Department of Gastroenterology, Xiangya Hospital, Central South University, Changsha, Hunan, China; 3 School of Nursing, Southwest Medical University, Luzhou, Sichuan, China

Removal of esophageal foreign bodies is a common emergency endoscopic treatment 1 . However, large foreign bodies and sharp edges are extremely risky and difficult to remove endoscopically, and affected patients often undergo surgical operations, which greatly increases the damage to the body 2 . We have developed an endoscopic anterior cap with an airbag specifically for the removal of these difficult foreign bodies ( Fig. 1 ), with excellent results.

**Fig. 1:**
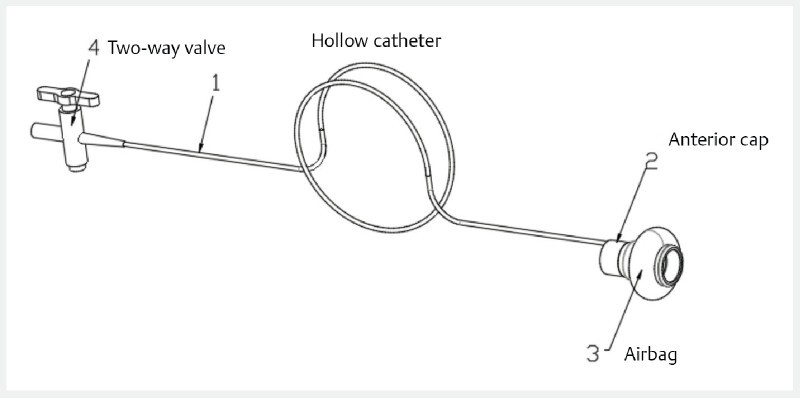
The endoscopic anterior cap with airbag, specifically for the removal of difficult foreign bodies.

A 61-year-old man had retrosternal pain after eating fish. Computed tomography showed a foreign body in the upper esophagus, 14 × 4 mm in size, which was embedded in the esophageal wall, adjacent to the left subclavian artery ( Fig. 2 ). As the foreign body was large, sharp, deeply embedded, and close to a blood vessel, it could not be removed by conventional endoscopic procedures, even with a transparent cap. We replaced the anterior cap with an airbag, entered the esophagus near the foreign body, inflated the airbag with about 20 mL of gas, and the expanded airbag effectively supported the esophageal wall and improved the operating space ( Fig. 3 ). We used foreign body forceps to lift the foreign body away from the esophageal wall, adjusted the orientation, and collected the foreign body into the anterior cap ( Fig. 4 ). After extracting the gas from the airbag, the foreign body was successfully removed ( Fig. 5 ). On examination, only mild mucosal lesions were present.

**Fig. 2:**
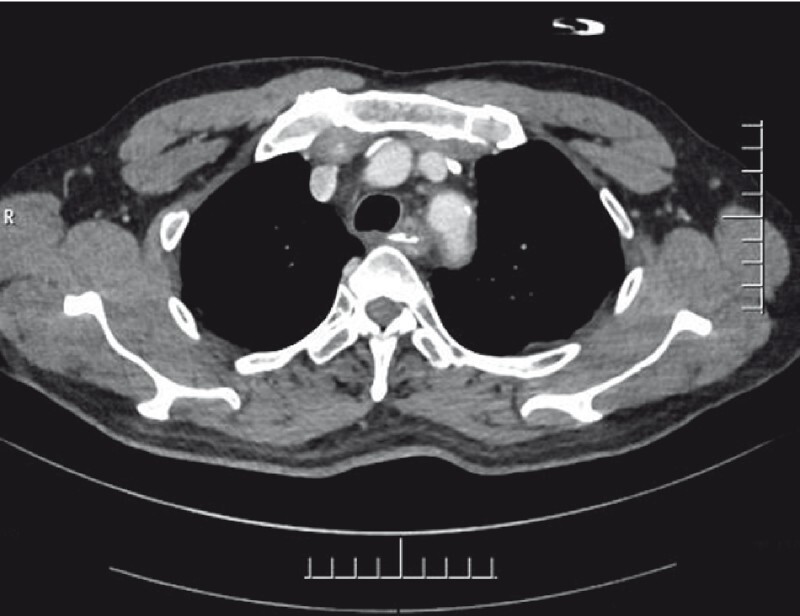
The foreign body in the upper esophagus, 14 × 4 mm in size, embedded in the esophageal wall.

**Fig. 3:**
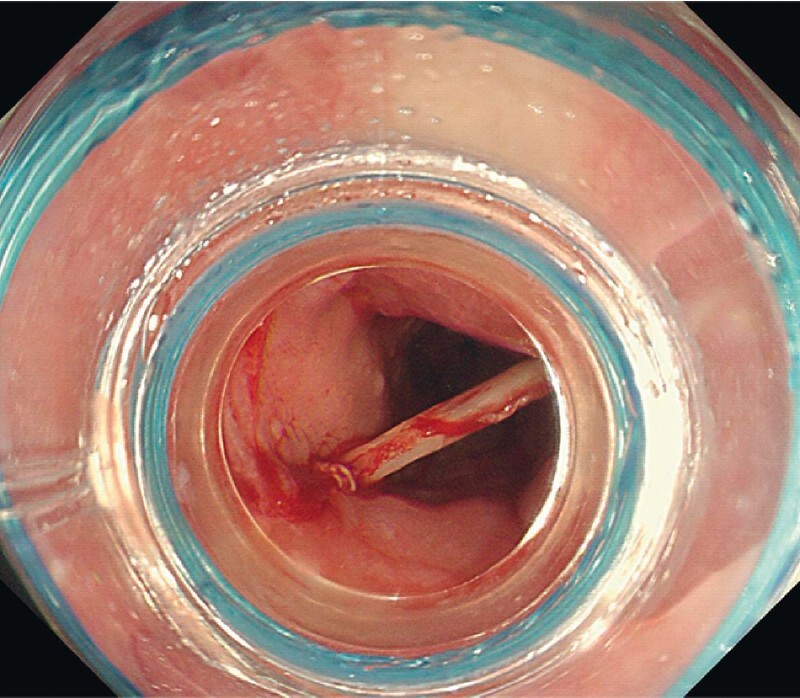
The expanded airbag effectively supported the esophageal wall and improved the operating space.

**Fig. 4:**
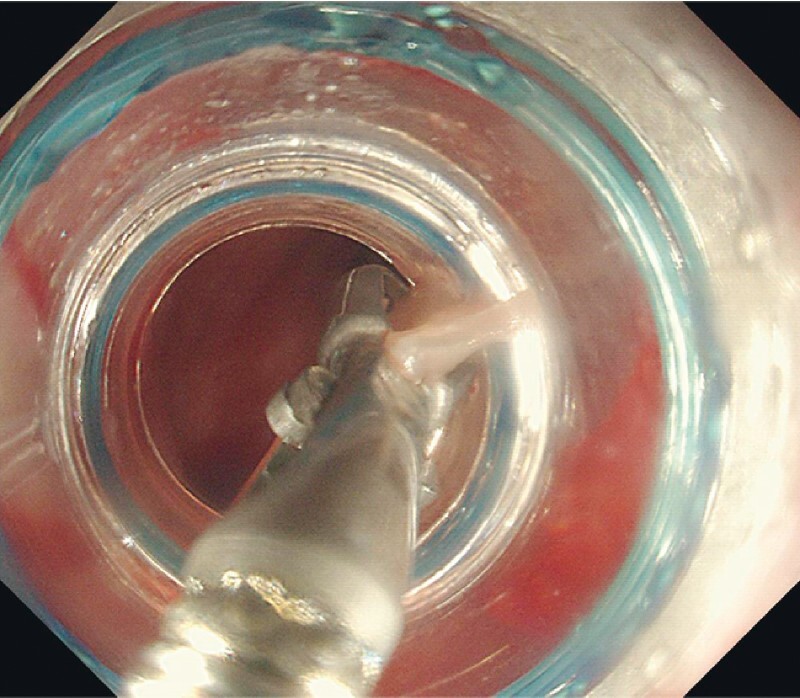
The foreign body was collected into the anterior cap.

**Fig. 5:**
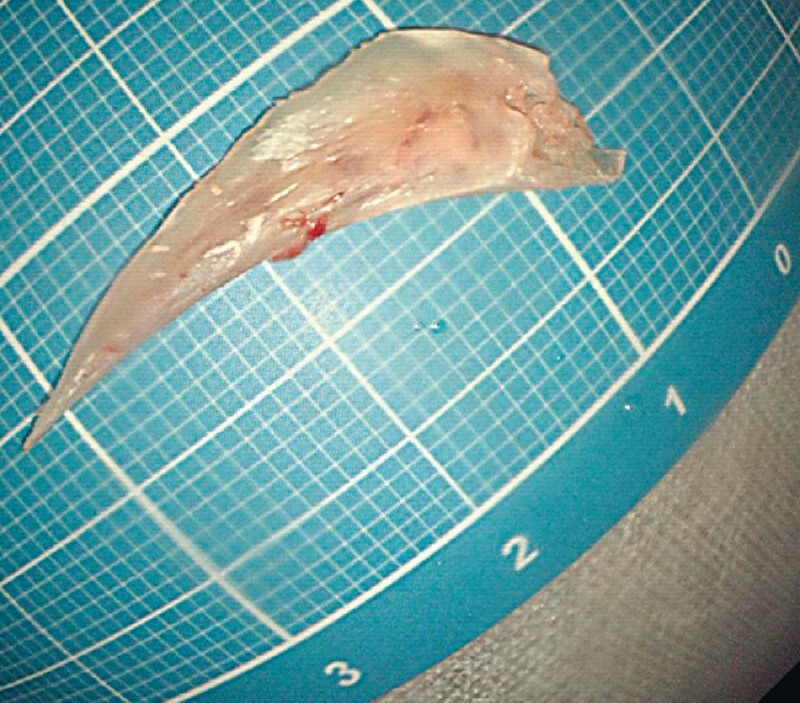
The foreign body was successfully removed.

The use of the airbag cap is shown in the video ( Video 1 ). A syringe is used to inflate the airbag, which can be expanded into a ring balloon with a maximum diameter of 3 cm, and the inflation amount is adjusted according to the needs of the operation. This method can effectively support the esophageal wall, improve the operating space, shorten the endoscopic surgery time, and reduce damage to the digestive tract. Further studies with large sample sizes are required to evaluate the safety and effectiveness of the airbag cap.

**Video 1** An airbag cap specially designed for the removal of sharp, deeply embedded foreign bodies.


Endoscopy_UCTN_Code_TTT_1AO_2AL

## Notes

Endoscopy E-Videos

https://eref.thieme.de/e-videos
*E-Videos* is an open access online section of the journal *Endoscopy* , reporting on interesting cases and new techniques in gastroenterological endoscopy. All papers include a high-quality video and are published with a Creative Commons CC-BY license. Endoscopy E-Videos qualify for HINARI discounts and waivers and eligibility is automatically checked during the submission process. We grant 100% waivers to articles whose corresponding authors are based in Group A countries and 50% waivers to those who are based in Group B countries as classified by Research4Life (see: https://www.research4life.org/access/eligibility/ ). This section has its own submission website at https://mc.manuscriptcentral.com/e-videos

## References

[1] Ikenberry S O Jue T L Anderson M A Management of ingested foreign bodies and food impactions Gastrointest Endosc 2011 73 1085 1091 21628009 10.1016/j.gie.2010.11.010

[2] Geng C Li X Luo R Endoscopic management of foreign bodies in the upper gastrointestinal tract: a retrospective study of 1294 cases Scand J Gastroenterol 2017 52 1286 1291 28691540 10.1080/00365521.2017.1350284

